# Metabolomic profiling reveals the step-wise alteration of bile acid metabolism in patients with diabetic kidney disease

**DOI:** 10.1038/s41387-024-00315-0

**Published:** 2024-10-09

**Authors:** Qing Zhang, Liqian Lu, Jiao Wang, Manman Lu, Dongwei Liu, Chunyu Zhou, Zhangsuo Liu

**Affiliations:** 1 Department of Integrated Traditional and Western Nephrology, The First Affiliated Hospital of Zhengzhou University, Zhengzhou, 450052 China; 2 Henan Province Research Center for Kidney Disease, Zhengzhou, 450052 China; 3 Blood Purification Center, The First Affiliated Hospital of Zhengzhou University, Zhengzhou, 450052 China

**Keywords:** Diabetic nephropathy, Diabetes complications

## Abstract

**Background:**

Diabetic kidney disease (DKD) is the major complication of diabetes concomitant with gut dysbiosis and glycometabolic disorder, which are strongly associated with bile acid (BA) metabolism. Yet studies investigating the BA metabolism involving in DKD pathogenesis are limited. This study aimed to explore the metabolomic profiling of BAs in DKD and analyze its association with DKD progression.

**Methods:**

An ultra-performance liquid chromatography tandem mass spectrometry (UPLC-MS/MS) method was established to quantify BAs in the plasma, fecal and urine samples of patients with DKD or T2DM and healthy individuals (*n* = 30 for each group). The key BAs associated with DKD were identified by orthogonal partial least-squares discriminant analysis (OPLS-DA) and receiver-operating characteristic (ROC) curve. Polynomial regression and Pearson’s correlation analyses were performed to assess the correlation between the key BAs and the clinical indicators reflecting DKD progression.

**Results:**

Metabolomic profiling of 50 kinds of BAs presented the markedly step-wise alterations of BAs in plasma and feces as well as the little in urine of patients with DKD. Eight kinds of BAs in the plasma, eight kinds in the feces and three kinds in the urine were abnormally expressed, accompanying with the increased conjugated/unconjugated ratios of cholic acid, deoxycholic acid, chenodeoxycholic acid, ursodeoxycholic acid and hyocholic acid in the plasma, and of cholic acid, chenodeoxycholic acid and lithocholic acid in the feces. Moreover, the increased plasma level of glycochenodeoxycholic acid, and the increased fecal levels of glycolithocholic acid, 7-ketodeoxycholic acid and chenodeoxycholic acid-3-β-D-glucuronide are strongly correlated with the clinical indicators reflecting DKD progression, including eGFR, 24 h urinary protein and 24 h urinary microalbumin.

**Conclusions:**

Our study for the first time disclosed the specific alterations of BA metabolism reflecting the step-wise progression of DKD, providing the basis for early identification and therapeutical strategies for DKD.

## Introduction

Of the long-term complications of diabetes, diabetic kidney disease (DKD) imposes the highest burden, both in terms of financial cost and the effects on daily life [1]. The presence of DKD leads to an increased risk for adverse health outcomes, including frailty, reduced quality of life, end-stage renal disease (ESRD), and premature mortality. Previous studies reported the importance of early intensive glycemic control to reduce risk factor of DKD. However, even after hyperglycemia has been brought under control, the “metabolic memory”, a cluster of irreversible metabolic changes, that allow the occurrence of DKD [2]. Therefore, a comprehensive understanding of metabolic control, in particular glucose and lipid metabolism, is essential to intervene aggressively, by means other than insulin administration or proteinuria reduction, to slow the progression of established DKD.

Bile acids (BAs) are cholesterol-derived metabolites that facilitate the intestinal nutrient absorption and biliary secretion of lipids, toxic metabolites, and xenobiotics. They also emerged as pivotal signaling molecules and metabolic regulators leading to regulation of intestinal incretin, hepatic gluconeogenesis, glycogen synthesis, energy expenditure, inflammation, and gut microbiome configuration [3]. Disorders of BA metabolism are associated with obesity and T2DM, whereas treating of patients with T2DM using BA sequestrants results in a significant improvement in glycemic response [4]. Moreover, BA accumulation in the plasma and its accompanying oxidative stress have been proposed as pathogenic factors of kidney injury [5]. However, studies exploring the step-wise changes of BA metabolism from healthy status to T2DM and then to DKD are limited.

Here, an ultra-performance liquid chromatography-tandem mass spectrometry (UPLC-MS/MS) was performed to simultaneously quantify representative BAs in the plasma, fecal and urine samples of patients with T2DM or DKD and healthy individuals, and to investigate the alterations of BA metabolism among them. Our results provide a basis for the full understanding of the pathogenic mechanisms of DKD and for prophylactic and therapeutic approaches based on the changes in BA metabolism.

## Methods

### Study design and participants

This study was approved by the Ethics Committee of the First Affiliated Hospital of Zhengzhou University (2020-KY-363) and complied with the Declaration of Helsinki regarding the ethical conduct of research involving human subjects. Written and informed consents to approve the use of blood, fecal and urine samples were obtained from all participants. Patients with DKD (DKD group) and patients with T2DM (T2DM group) were recruited from the Department of Nephrology and Department of Endocrinology respectively, while healthy individuals (CON group) were recruited from the Department of Physical Examination, of the First Affiliated Hospital of Zhengzhou University from January. The healthy individuals were sex- and age-matched to the patients with DKD and with T2DM at a statistical significance level >0.05. The inclusion and exclusion criteria for patients with DKD, patients with T2DM, and healthy individuals were as described previously [6]. Estimated glomerular filtration rate (eGFR) was calculated using MDRD equation [7]. Plasma, urine and fecal samples from CON, T2DM, and DKD groups (30 patients in each group) were collected from January 2022 to December 2022.

### Sample collection and preparation

Blood samples were collected from the participants in the morning after overnight fasting with an ethylene diamine tetraacetic acid (EDTA) anti-coagulant tube. The plasma samples were then separated with centrifugation at 1500 × g for 10 min at 4 °C within 1 h after collection. Fecal samples were freshly collected in the morning after overnight fasting. Midstream urine samples were collected from the participants in the morning after overnight fasting and centrifuged at 12,000 × g for 10 min at 4 °C within 1 h after collection. All plasma, fecal and urine samples were then immediately frozen at −80 °C until further analysis.

Stock solutions of the 50 kinds of BAs were prepared by dissolving standard compounds (MedChem Express, Monmouth Junction, NJ, United States) in water at concentrations and stored in brown volumetric flasks at −80 °C until use (Supplementary Table 1). Isotope-labeled mix of 9 kinds of BAs was diluted with methanol at a concentration of 100 ng/mL to act as internal standard (IS). Working solutions of BAs were prepared by diluting stock solutions into twelve different batches (0.1–1000 ng/mL) with methanol.

Plasma, fecal and urine samples were prepared using a protein precipitation extraction method. Briefly, 50 μL of thawed plasma or urine, 50 μL IS and 200 μL methanol were transferred into a 1.5 mL tube. As regard to feces, 20 mg fecal samples were mixed with 50 μL IS and 200 μL methanol and then homogenated at 4 °C for 10 min. Afterwards the above plasma, fecal and urine mixes were vortexed at 2500 rpm for 10 min and put at −20 °C for 10 min. After centrifugation at 12,000 rpm for 10 min at 4 °C, the supernatant was evaporated completely at 30 °C using a centrifugal vacuum evaporator and then redissolved in 100 μL 50% methanol/water (V/V) for further UPLC-MS/MS analysis.

### UPLC-MS/MS-based BAs detection

The reconstituted samples were loaded onto a Waters ACQUITY UPLC HSS T3 C18 column (100 mm × 2.1 mm, 1.8 μm particle size; Waters, Milford, MA, USA) for chromatographic separation. The isocratic gradient elution program was run with mobile phase A (water with 0.01% acetic acid and 5 mM ammonium acetate) and mobile phase B (acetonitrile with 0.01% acetic acid, Supplementary Table 2). The BAs were then detected by AB 6500 + QTRAP LC-MS/MS System (Applied Biosystems Sciex, Toronto, Canada), equipped with an ESI Turbo Ion-Spray Interface, operating in negative ion modes and controlled by Analyst 1.6.3 software. The optimized MS conditions for BA detection are presented in Supplementary Table 3, while the detailed declustering potential, collision energy, precursor and dominant daughter ions of the BAs and ISs, are listed in Supplementary Table 4 and Supplementary Table 5. The chromatograms of BAs and IS were conducted by OriginLab (OriginLab, Northampton, MA, United States). The concentrations of the BAs in plasma, fecal and urine samples were calculated against the corresponding calibration curves with standards. The fecal BAs were then normalized by the sample weights.

### Metabolomic profiling of BAs

The BA levels in plasma, fecal and urine samples were imported into SIMCA-P v16.0.2 (Umetrics Suite; Sartorius, Umeå, Sweden). Orthogonal projections to latent structures discriminant analysis (OPLS-DA) was established to explore the discrimination between the DKD group, T2DM group and CON group. The quality of all OPLS-DA models was evaluated by the goodness-of-fit parameter (R^2^) and the predictive ability parameter (Q^2^). The score plot was performed to visualize the discrimination ability of the OPLS-DA model. OPLS-DA1 represents the predictive component, which indicates the difference between groups, while OPLS-DA2 represents the orthogonal components, which indicates the difference within groups. The accompanying shared and unique structures (SUS) plot and variable importance in projection (VIP) analyses were generated to determine the major latent BAs in the data matrix and contributions of each BA to the group discrimination. VIP value > 1.2 was defined as statistical significance in terms of discriminating between groups.

### Statistical analysis

Continuous data in normal distribution were expressed as mean ± standard deviation, while the data in non-normal distribution were presented as median (quartile). Two-tailed *t*-test, Kruskal-Wallis test followed by Bonferroni post hoc comparison test (abnormal distributed data), and one-way ANOVA followed by Tukey’s (normal distributed data with equal variances), or Games-Howell (abnormal distributed data with unequal variances) using Prism v8.0.2 software (GraphPad, San Diego, CA, USA) with *P* < 0.05 as the level of significance. Polynomial regression analyses and Pearson’s correlation coefficients were performed to assess the correlation between the significantly-altered BAs in the plasma, fecal and urine samples and with the clinical indicators, respectively. R^2^ > 0.7 was considered as of noticeable correlativity. Receiver-operating characteristic (ROC) curves were performed with SPSS v21.0 (Armonk, NY, United States). The corresponding area under the ROC curve (AUC) was conducted to evaluate the predictive performances of the key BAs for DKD with AUC > 0.7 as of the significance.

## Results

### Metabolomic profiling of BAs in plasma, urine and fecal samples

**Fig. 1:**
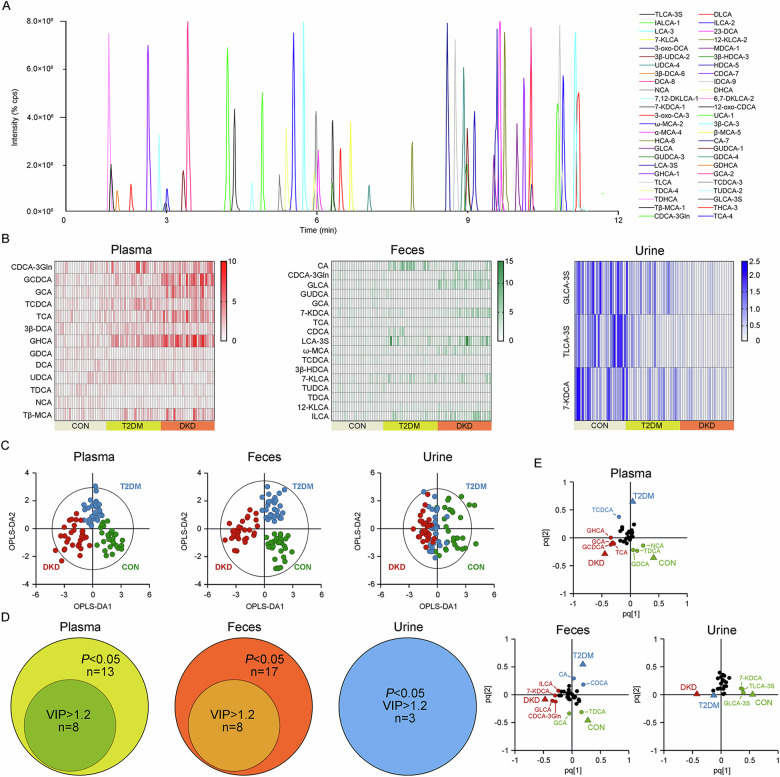
Plasma, fecal and urine profiling of BAs in the step-wise alterations of DKD patients. **A** Chromatogram of 50 BAs in MRM mode. **B** The heatmap of preliminarily selected differential metabolites in the plasma, feces and urine samples of the DKD, T2DM and CON groups. **C** The OPLS-DA analyses of the metabolomic profiling of the plasma, feces and urine samples from the DKD, T2DM and CON groups. **D** The Venn diagrams for overlap of key BAs with *P* < 0.05 and VIP value > 1.2 in the plasma, feces and urine samples of the DKD, T2DM and CON groups. **E** The loading plots of the key BAs with both *P* < 0.05 and VIP value > 1.2 in the plasma, feces and urine samples of the DKD, T2DM and CON groups. The detailed information relating to the chromatogram of each BA is listed in Supplementary Table S4.

The demographic characteristics of the study population were extracted from the medical record system of hospital and are presented in Supplementary Table 6. Of 50 kinds of BAs in the established UPLC-MS/MS method (Fig. 1A), twenty-three kinds in the plasma, twenty kinds in the feces, and twenty-nine kinds in the urine, with >30% of the measurements below the lower limit of quantitation (LOQ), were excluded. Within the detected BAs, thirteen kinds in the plasma, seventeen kinds in the feces, and three kinds in the urine were significantly altered in DKD group than in T2DM group or CON group (Fig. 1B).

The OPLS-DA analysis was then performed, highlighting the optimal discriminations among the three groups in the plasma (Fig. 1C; R^2^X = 0.366, R^2^Y = 0.724, Q^2^ = 0.607) and feces (R^2^X = 0.392, R^2^Y = 0.787, Q^2^ = 0.675), yet non-optimal discrimination in the urine (R^2^X = 0.239, R^2^Y = 0.356, Q^2^ = 0.285), with optimal binary classifier, validity and degree of overfitting (Supplementary Fig. 1). According to the significance of *P* < 0.05 in the Student’s t-test and VIP > 1.2 in the OPLS-DA model, eight kinds of BAs, including Glycochenodeoxycholic acid (GCDCA), Glycohyocholic acid (GHCA), Glycocholic acid (GCA), Taurocholic acid (TCA), Taurochenodeoxycholic acid (TCDCA), Glycodeoxycholic acid (GDCA), Taurodeoxycholic acid (TDCA), and Norcholic acid (NCA), were the key BAs in the plasma contributing to the group difference among three groups (Fig. 1D, E). Eight kinds of BAs in the feces, including TDCA, Glycolithocholic acid (GLCA), GCA, Cholic acid (CA), Chenodeoxycholic acid-3-β-D-glucuronide (CDCA-3Gln), 7-Ketodeoxycholic acid (7-KDCA), Chenodeoxycholic acid (CDCA) and Isolithocholic acid (ILCA), as well as Taurolithocholic acid-3-sulfate (TLCA-3S), Glycolithocholic acid-3-sulfate (GLCA-3S) and 7-KDCA in the urine, were the major BAs contributing to the group differences.

### The separated metabolomic profiling of plasma BAs

**Fig. 2:**
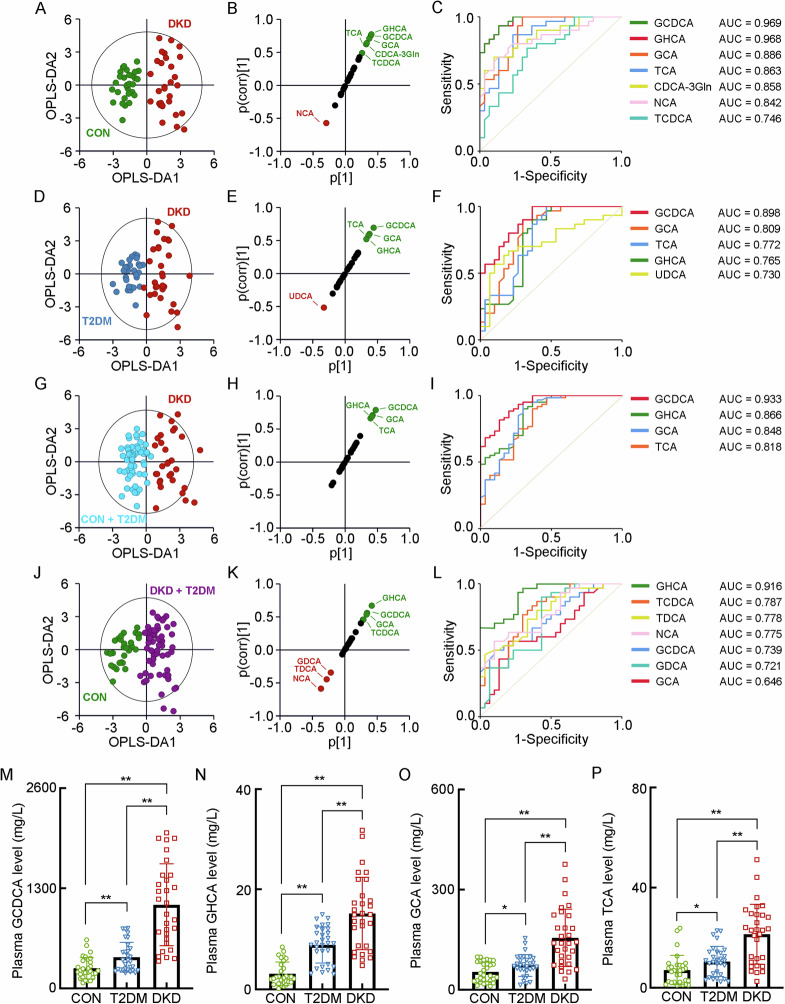
The separated metabolomic profiling of the key BAs in the plasma of the patients with DKD and T2DM, and healthy individuals. **A** The OPLS-DA score plot between the DKD group and the CON group. The SUS-plot (**B**) and ROC curve analyses (**C**) of the key BAs between the DKD group and the CON group. **D** The OPLS-DA score plot between the DKD group and the T2DM group. The SUS-plot (**E**) and ROC curve analyses (**F**) of the key BAs between the DKD group and the T2DM group. **G** The OPLS-DA score plot between the DKD group and the Non-DKD group (both T2DM group and CON group). The SUS-plot (**H**) and ROC curve analyses (**I**) of the key BAs between the DKD group and the Non-DKD group. **J** The OPLS-DA score plot between the group in diabetic condition (both DKD and T2DM group) and the CON group. The SUS-plot (**K**) and ROC curve analyses (**L**) of the key BAs between the group in diabetic condition and CON group. The plasma levels of the key BAs, including **M** GCDCA, **N** GHCA, **O** GCA, **P** TCA. *P < 0.05, **P < 0.01.

For the BA profiling in plasma, six kinds of the primary conjugated BAs, including CDCA-3Gln, GCDCA, GCA, TCDCA, TCA and NCA, and four kinds of the secondary conjugated BAs, including GHCA, GDCA, ursodeoxycholic acid (UDCA) and TDCA, as well as two kinds of the secondary unconjugated BAs, namely deoxycholic acid (DCA) and 3β-deoxycholic acid (3β-DCA), were significantly altered in the patients with DKD (Supplementary Table 7). When exploring the detailed BA metabolism in the patients with T2DM or DKD, or patients in the diabetic condition (either T2DM or DKD), or patients in Non-DKD condition (either CON or T2DM), the OPLS-DA model was employed, visualizing the optimal separation between the DKD group and CON group (R^2^X = 0.365, R^2^Y = 0.866, Q^2^ = 0.761, Fig. 2A). The SUS-plots and ROC analyses identified GCDCA, GHCA, GCA, TCA, CDCA-3Gln, NCA and TCDCA as key BAs discriminating the DKD group from CON group (Fig. 2B, C). The OPLS-DA analysis also visualized the separation between the DKD group and T2DM group (R^2^X = 0.339, R^2^Y = 0.771, Q^2^ = 0.589, Fig. 2D). The SUS-plots and ROC analysis identifying GCDCA, GCA, TCA, GHCA and UDCA as key BAs discriminating the DKD group from T2DM group (Fig. 2E, F). In addition, it also indicated that GCDCA, GHCA, GCA and TCA were the key BAs discriminating the DKD group from the Non-DKD group (R^2^X = 0.315, R^2^Y = 0.784, Q^2^ = 0.675, Fig. 2G–I), while the GHCA, TCDCA, TDCA, NCA, GCDCA, GDCA, and GCA were the key BAs identifying the patients in diabetic condition from the CON group (R^2^X = 0.231, R^2^Y = 0.771, Q^2^ = 0.677, Fig. 2J−L). Accompanied with the significant alteration of these six kinds of BAs in plasma (Fig. 2M–R), the combined results indicated that GCDCA, GCA, TCA and GHCA were significantly increased in the DKD group and strongly correlated with the progression of DKD from either T2DM or healthy status.

### The separated metabolomic profiling of fecal BAs

**Fig. 3:**
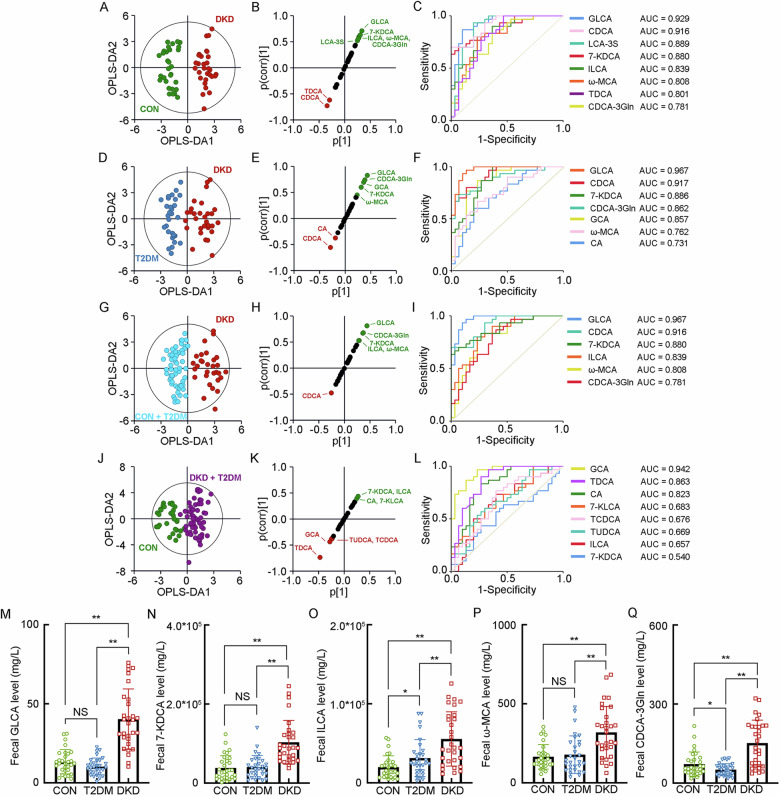
The separated metabolomic profiling of the key BAs in the feces of the patients with DKD and T2DM, and healthy individuals. **A** The OPLS-DA score plot between the DKD group and the CON group. The SUS-plot (**B**) and ROC curve analyses (**C**) of the key BAs between the DKD group and the CON group. **D** The OPLS-DA score plot between the DKD group and the T2DM group. The SUS-plot (**E**) and ROC curve analyses (**F**) of the key BAs between the DKD group and the T2DM group. **G** The OPLS-DA score plot between the DKD group and the Non-DKD group (both T2DM group and CON group). The SUS-plot (**H**) and ROC curve analyses (**I**) of the key BAs between the DKD group and the Non-DKD group. **J** The OPLS-DA score plot between the group in diabetic condition (both DKD and T2DM group) and the CON group. The SUS-plot (**K**) and ROC curve analyses (**L**) of the key BAs between the group in diabetic condition and CON group. The levels of the key fecal BAs, including **M** GLCA, **N** 7-KDCA, **O** ILCA, **P** ω-MCA, and **Q** CDCA-3Gln. *P < 0.05, **P < 0.01, NS, no significance.

For the BA profiling in feces, the significant alterations of seventeen kinds of BAs presented in the DKD group, including (1) two kinds of primary unconjugated BAs, namely CA and CDCA, (2) five kinds of primary conjugated BAs, including TCA, TCDCA, CDCA-3Gln, GCDCA and ω-Muricholic acid (ω-MCA), (3) four kinds of secondary unconjugated BAs, namely 7-Ketolithocholic acid (7-KLCA), 12-KLCA, ILCA and 7-KDCA, and (4) six kinds of secondary conjugated BAs, including 3β-Hyodeoxycholic acid (3β-HDCA), LCA-3S, Tauroursodeoxycholic acid (TUDCA), TDCA, GLCA and Glycoursodeoxycholic acid (GUDCA) (Supplementary Table 8). The separated OPLS-DA analysis showed the optimal separation between the DKD group and the CON group (R^2^X = 0.291, R^2^Y = 0.915, Q^2^ = 0.868, Fig. 3A), with GLCA, CDCA, LCA-3S, 7-KDCA, ILCA, ω-MCA, TDCA and CDCA-3Gln as the key BAs to group difference (Fig. 3B, C). The OPLS-DA analysis between the DKD group and the T2DM group (R^2^X = 0.274, R^2^Y = 0.827, Q^2^ = 0.723, Fig. 3D) also showed the ideal modeling with GLCA, CDCA, 7-KDCA, CDCA-3Gln, GCA, ω-MCA and CA as the key BAs contributing to the group difference (Fig. 3E, F). In addition, it also indicated that GLCA, CDCA, 7-KDCA, ILCA, ω-MCA and CDCA-3Gln as the key BAs discriminating the DKD group from the Non-DKD group (R^2^X = 0.251, R^2^Y = 0.833, Q^2^ = 0.759, Fig. 3G–I), while GCA, TDCA, CA, 7-KLCA, TCDCA, TUDCA, ILCA and 7-KDCA were the key BAs identifying the patients in diabetic condition from the CON group (R^2^X = 0.246, R^2^Y = 0.779, Q^2^ = 0.705, Fig. 3J–L). Combing the significant increase of their fecal levels, GLCA, 7-KDCA, ILCA, ω-MCA and CDCA-3Gln were positively correlated with the progression of DKD from either T2DM or healthy status (Fig. 3M–Q).

### The separated metabolomic profiling of urinary BAs

**Fig. 4:**
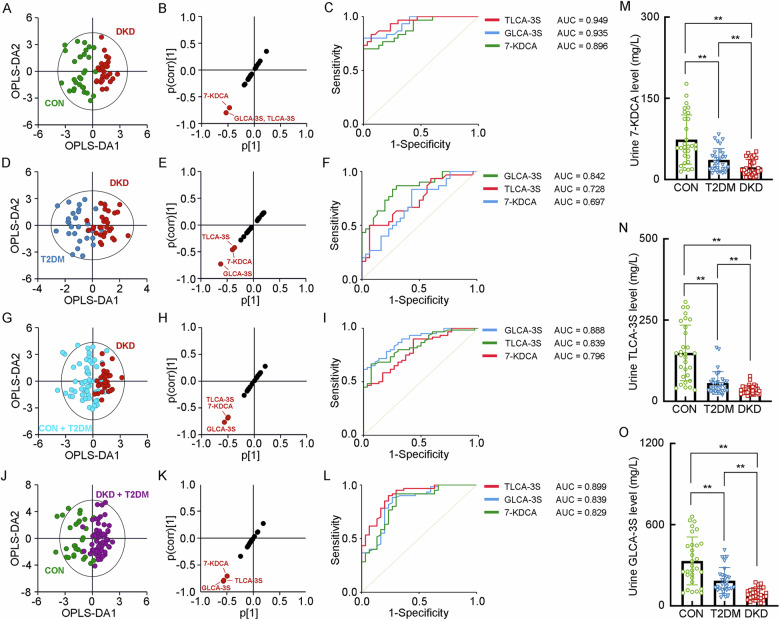
The separated metabolomic profiling of the key BAs in the urine samples of the patients with DKD and T2DM, and healthy individuals. **A** The OPLS-DA score plot between the DKD group and the CON group. The SUS-plot (**B**) and ROC curve analyses (**C**) of the key BAs between the DKD group and the CON group. **D** The OPLS-DA score plot between the DKD group and the T2DM group. The SUS-plot (**E**) and ROC curve analyses (**F**) of the key BAs between the DKD group and the T2DM group. **G** The OPLS-DA score plot between the DKD group and the Non-DKD group (both T2DM group and CON group). The SUS-plot (**H**) and ROC curve analyses (**I**) of the key BAs between the DKD group and the NonDKD group. **J** The OPLS-DA score plot between the group in diabetic condition (both DKD and T2DM group) and the CON group. The SUS-plot (**K**) and ROC curve analyses (**L**) of the key BAs between the group in diabetic condition and CON group. The levels of the key fecal BAs, including **M** 7-KDCA, **N** TLCA-3S and **O** GLCA-3S. *P < 0.05, **P < 0.01.

When analyzing the BA levels in the urine, it presented the decrease of GLCA-3S, TLCA-3S and 7-KDCA in the DKD group (Supplementary Table 9). The OPLS-DA models showed the poor performance between the DKD group and CON group (R^2^X = 0.248, R^2^Y = 0.754, Q^2^ = 0.591), the DKD group and T2DM group (R^2^X = 0.179, R^2^Y = 0.593, Q^2^ = 0.326), the DKD group and Non-DKD group (R^2^X = 0.234, R^2^Y = 0.451, Q^2^ = 0.271), and the patients in diabetic condition from the CON group (R^2^X = 0.237, R^2^Y = 0.672, Q^2^ = 0.529), with the validation of the significance of TLCA-3S, GLCA-3S, and 7-KDCA in urine that contribute to the DKD identification by the SUS-plots and ROC analyses (Fig. 4A–L). It also showed step-wise decrease of these three BAs in the progression of DKD from T2DM and CON. These results indicated that the urine GLCA-3S, TLCA-3S and 7-KDCA may significantly involve in the progression of DKD (Fig. 4M–O).

### The ratios between conjugated- and unconjugated- BAs

The ratios between conjugated BAs and unconjugated BAs detected in the plasma, feces and urine of patients with T2DM, patients with DKD and healthy individuals, were calculated, showing the increased conjugated/unconjugated ratios of CA, DCA, CDCA, UDCA, and HCA in the plasma, and of CA, CDCA and LCA in the feces (Table 1). These findings suggested that the patients with DKD have increased transition from unconjugated- to conjugated- BAs.

**Table 1:** The calculated ratios between some conjugated and unconjugated bile acids in the study participants.

Conjugated/Unconjugated	CON (*n* = 30)	T2DM (*n* = 30)	DKD (*n* = 30)	P*-value	*P-*value		
					T2DM vs CON	DKD vs CON	DKD vs T2DM
**Plasma**							
CA	2.24 ± 1.98	2.47 ± 1.24	4.28 ± 3.08	< 0.01	0.59	< 0.01	< 0.01
DCA	2.22 ± 1.81	1.00 ± 0.78	2.08 ± 1.83	< 0.01	< 0.01	0.77	< 0.01
CDCA	0.55 ± 0.41	1.03 ± 0.88	2.31 ± 1.94	< 0.01	< 0.01	< 0.01	< 0.01
UDCA	0.53 ± 0.38	0.31 ± 0.22	0.82 ± 0.59	< 0.01	0.01	0.03	< 0.01
LCA	0.48 (1.35)	0.56 (2.38)	0.56 (0.81)	0.21	0.03	0.36	< 0.01
HCA	0.27 (0.64)	0.36 (0.91)	1.34 (2.01)	< 0.01	0.03	0.36	< 0.01
**Feces**							
CA	0.88 ± 0.86	0.15 ± 0.15	0.38 ± 0.27	< 0.01	< 0.01	< 0.01	< 0.01
DCA	0.02 ± 0.01	0.02 ± 0.02	0.02 ± 0.01	0.91	0.84	0.79	0.68
CDCA	0.03 (0.02)	0.02 (0.02)	0.11 (0.23)	< 0.01	0.03	< 0.01	< 0.01
UDCA	0.099 (0.026)	0.004 (0.004)	0.008 (0.073)	0.39	0.58	< 0.01	< 0.01
LCA	0.0007 (0.0015)	0.0011 (0.0239)	0.0018 (0.0040)	< 0.01	< 0.01	< 0.01	< 0.01
**Urine**							
CA	1.54 ± 1.10	1.49 ± 1.26	1.19 ± 1.06	0.44	0.87	0.21	0.32
DCA	0.74 (0.80)	0.99 (1.69)	0.95 (2.27)	< 0.01	< 0.01	< 0.01	< 0.01
CDCA	1.83 (2.30)	1.35 (4.21)	1.70 (7.80)	0.39	< 0.01	0.47	< 0.01
LCA	0.70 (1.36)	0.62 (0.83)	1.11 (1.55)	0.22	< 0.01	0.11	< 0.01

Continuous data in normal distribution were expressed as mean ± standard deviation, while the data in non-normal distribution were presented as median (quartile). P* values were determined by one-way analysis of variance, while *P*-values between sub-groups were determined by Student’s *t*-test. CON, healthy controls; *T2DM* type 2 diabetes mellitus; *DKD* diabetic kidney disease.

### The correlation matrix of BAs

**Fig. 5:**
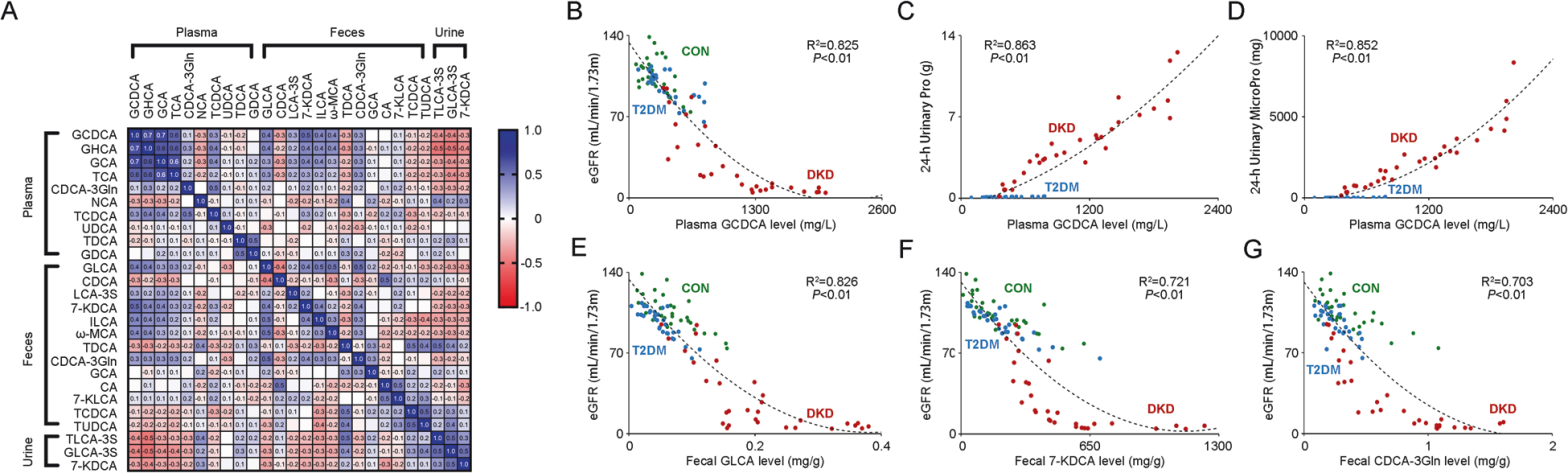
Correlation matrix for the BAs in the study participants. **A** The correlation heat map of the key BAs in the plasma, fecal and urine samples of the study participants. The blue area shows strong positive correlation, the red area shows strong negative correlation. The specific Pearson’s r value is presented in each cell. The cells in white were defined as *R*=0. Scatter plots show the correlation of plasma levels of GCDCA with **B** eGFR, **C** 24-h urinary protein, and **D** 24-h urinary microprotein. Scatter plots show the correlation of eGFR with the fecal levels of **E** GLCAA, **F** 7-KDCA, and **G** CDCA-3Gln. The green dots, blue dots and red dots represent the samples in the CON group, T2DM group and DKD group, respectively.

The correlation matrix for BAs in study participants were performed, showing the positive correlation of the key BAs in the feces and the negative correlation of the key BAs in the urine with the key BAs in the plasma. Moreover, it also presented the strong correlation of plasma GCDCA, GHCA and GCA between each other (R^2^ > 0.7, Fig. 5A). When analyzing their correlation with the clinical indicators reflecting DKD progression, including hemoglobin, serum albumin, serum creatinine, blood glucose, serum triglyceride, total cholesterol, low-density lipoprotein, high-density lipoprotein, parathyroid hormone, glycated hemoglobin, eGFR and urinary protein and urinary microalbumin in 24 h, it presented the strong correlation of GCDCA with eGFR (R^2^ = 0.825, Fig. 5B), 24 h urinary protein (R^2^ = 0.863, Fig. 5C) and 24 h urinary microalbumin (R^2^ = 0.852, Fig. 5D). In addition, the fecal levels of GLCA, 7-KDCA and CDCA-3Gln also had the strong correlations with eGFR (R^2^ > 0.7, Fig. 5E–G). These results indicated the optimal correlation of BAs within the plasma, feces and urine, and the levels of GCDCA in plasma, and the levels of GLCA, 7-KDCA and CDCA-3Gln in the feces were the key BAs reflecting the impairment of renal function in patients with DKD.

## Discussion

Diabetes remains a key reason for developing ESRD and cause about one third of patients requiring renal replacement therapy [8]. BAs are considered to be important enteroendocrine hormone-like signaling molecules that regulate glucose, lipid, and energy metabolism. More importantly, timely identification of the altered BA metabolism associated with the impaired renal function should allow us not only to pinpoint individuals at the highest risk of developing ESRD, but to improve our mechanistic and clinical knowledge of DKD. To date, whilst the convincing evidence presented the increased BA pool size in the diabetic mice, it is still uncertain whether the alterations of BA metabolism in patients with T2DM, and much less concern for patients with DKD. Interestingly, conflicting results existed against the experimental evidence in the current available human studies [9]. A small cross-sectional study reported that the fasting taurine-conjugated BA concentrations were higher in patients with T2DM [10]. Another observational study involving 1707 patients with T2DM in Chinese population indicated the higher plasma levels of conjugated primary BAs and a secondary BA, were associated with an increased risk of T2DM [11]. Our study confirmed and expanded the previous observations by showing that the plasma levels of three primary conjugated BAs (GCDCA, GCA and TCA), and one secondary conjugated BA (namely GHCA), were significantly higher in patients with T2DM, and were much higher in patients with DKD. Together with the increased ratios between certain conjugated- and unconjugated- BAs in the plasma and feces of T2DM and DKD, our findings support the hypothesis that patients with T2DM and DKD have increased transition from unconjugated- to conjugated- BAs.

Notably, we presented the strong correlation of plasma GCDCA with eGFR, 24 h urinary protein and 24 h urinary microalbumin. GCDCA is one of the most toxic BAs which is originally synthesized in the liver by CDCA conjugated to glycine. The relatively high concentration of GCDCA in bile exerts hepatotoxic effect, which displays as the apoptosis of hepatocytes, the fibrotic disorder of hepatobiliary system, and the chemoresistance in hepatocellular carcinoma [12–14]. Thus, our study for the first time proposed the possible toxicity of GCDCA to DKD progression and indicated the clinical potential of GCDCA plasma concentration as the biomarker reflecting DKD progression, which also provide a new potential therapeutic target for DKD.

In addition to the plasma, the significant alterations of seventeen kinds of BAs in the feces of patients with DKD were also identified, most of which were increased. Under physiologic condition, only 5% of the total BAs is excreted into the feces owing to the enterohepatic circulation of BAs [15]. The diabetic intestinal dysfunction results in the decrease of intestinal BA reabsorption and thus excessive BA excretion in the feces [16]. Similarly, the unconjugated BAs were significantly increased in the feces of diabetic mice, while no conjugated BA was readily detected [17]. Treating with metformin could increase the BA resorption in the ileum by upregulating the expression of Cystic fibrosis transmembrane conductance regulator (CFTR) and Glucagon-like peptide-1 (GLP-1) [18]. Another study also proofed that the sleeve gastrectomy has the anti-diabetic benefits, partly due to the post-surgery overexpression of the BA transporter in the ileum [19]. In consistent with these evidence, our study showed the significant increase of ten kinds of BAs in the plasma, five of which were strongly correlated with the progression of DKD. The difference is that four kinds of conjugated BAs (GUDCA, TCA, TCDCA and TUDCA) were decreased in the fecal samples while increased in the plasma samples, of patients with either T2DM or DKD. Since most conjugated BAs are actively absorbed in the terminal ileum by the apical sodium-dependent bile acid transporter (ASBT), enhanced reabsorption of these conjugated BAs in our results may mainly attribute to the augmented activation of ASBT [20]. This hypothesis is also supported by the evidence that high levels of glucose contribute to the increased ASBT function in diabetes, while inhibiting ASBT could increase fecal excretion and improve glucose hemostasis [21]. Thus, the evidence above implicated that the BA excretion in the feces and reabsorption were both increased in patients with DKD, possibly due to the impaired ileum function and enhanced activation of renal BA transporter, respectively.

Finally, our study also presented the rare change of BA profiling in the urine of patients with T2DM and DKD. Although kidney is reported to play a minor role in BA excretion, there is evidence suggested that impairment in kidney function may profoundly affect BA homeostasis. Jimenez F et al. found that the BA excretion in the urine decreased significantly in patients with chronic renal failure (CRF), and also indicated that it could be enough to increase serum BA concentrations if the kidney function declined for a long enough period, despite of the small BA elimination rate by the kidney [22]. This alteration is probably due to not only the impaired renal BA excretion, but also the silent hepatobiliary alterations or an impaired BA enterohepatic circulation. However, at present there is a lack of evidence illustrating this issue in patients with CRF as well as DKD. It is still unclear whether the altered BA concentrations in plasma and feces of patients with DKD are primarily due to the fact that the role of the kidney in BA excretion is more important than previously thought. Further investigations are required to improve our current knowledge on the association between impaired kidney function and urinary BA excretion in patients with DKD.

Our study for the first time presenting the decreased urinary excretion of GLCA-3S, TLCA-3S and 7-KDCA in patients with T2DM or DKD.LCA is the only natural monohydroxy BA that formed in the large intestine by bacterial dehydroxylation of CDCA. Controversy exists regarding the pros or cons of LCA on living organisms, which mainly depends on the sulfation capacity in the different tissues [23]. Sulfation is an important detoxification pathway of LCA to enhance its fecal and urinary excretion [24]. Sulfated LCA are more rapidly excreted in the urine and is less efficiently reabsorbed in the intestine than non-sulfated lithocholate. Moreover, taurine and glycine conjugation increase the hydrophilicity to the LCA, enhancing its degree of detoxification [25]. Thus, the decreased urinary levels of GLCA-3S and TLCA-3S may reflect the enhanced detoxification and excretion of LCA in the step-wise progression from healthy individuals to T2DM and then to DKD.

Some limitations exist regarding to our study, First, since this is a cross-sectional study, there is insufficient evidence explicating the causality between BAs and DKD progression. Second, the sample size of our study was relatively small; the BA profiles at different stages of DKD were not sufficiently determined. Third, our study is a targeted BA metabolomic profiling including a total of 50 kinds of BAs, a combination of untargeted and targeted metabolomic research may possess a higher opportunity to clarify the alteration of BA profiles along with the DKD progression. Therefore, further investigations, including the prospective longitudinal study, in-vivo, or in-vitro intervention studies, are needed to explore the association between BA metabolism and DKD progression.

## Conclusions

Our study preliminarily presented the markedly step-wise alterations of BAs in plasma and feces as well as the little in urine in patients with DKD, and highlighted the increased transition from unconjugated- to conjugated- BAs. Moreover, we found that the correlation of increased plasma level of GCDCA, and the increased fecal levels of GLCA, 7-KDCA with the renal function in diabetes. However, the evidence is limited due to it being a single-center study with limited sample size. The association between BA metabolism, especially the plasma BA profiling, and the severity of kidney injury in the diabetic condition are not investigated. Expanded sample size is necessary in any further study. Moreover, the explicit mechanism regarding the increased BA transition from unconjugated- to conjugated- BAs, involves in the progression of DKD and whether therapeutic manipulation of these alterations can be kidney-beneficial remains to be explored. In summary, our study for the first time disclosed the specific alterations of BA metabolism reflecting the step-wise progression of DKD, which may provide the basis not only for the early identification but also the therapeutic strategies for DKD.

## Supplementary information

Supplementary Material

## Data Availability

The raw data generated or analyzed during this study are available from the corresponding author on reasonable request.
