# Supplementary material for: Metabolomic profiling reveals the step-wise alteration of bile acid metabolism in patients with diabetic kidney disease
Source: Nutr Diabetes. 2024 Oct 9;14:85. doi: 10.1038/s41387-024-00315-0 (PMC11464666; doi:10.1038/s41387-024-00315-0)
Supplement: Supplementary file 1 — Supplementary Material [file 41387_2024_315_MOESM1_ESM.docx]

**Supplementary materials for**

**Figure and Table of Contents**

**Supplementary Figure 1.** The validation of the OPLS-DA models.

**Supplementary Table 1.** The standard compounds of bile acids in our project.

**Supplementary Table 2.** Gradient program of the liquid chromatography mobile phase.

**Supplementary Table 3.** The optimized mass spectrometry conditions for the detection of bile acids.

**Supplementary Table 4.** The mass spectrometry parameters of the bile acids.

**Supplementary Table 5.** The mass spectrometry parameters of the isotoped-labelled bile acids.

**Supplementary Table 6.** General characteristics of the study population.

**Supplementary Table 7.** Plasma levels of the 27 bile acids in study participants.

**Supplementary Table 8.** Fecal levels of the 30 bile acids in study participants.

**Supplementary Table 9.** Urine levels of the 21 bile acids in study participants.


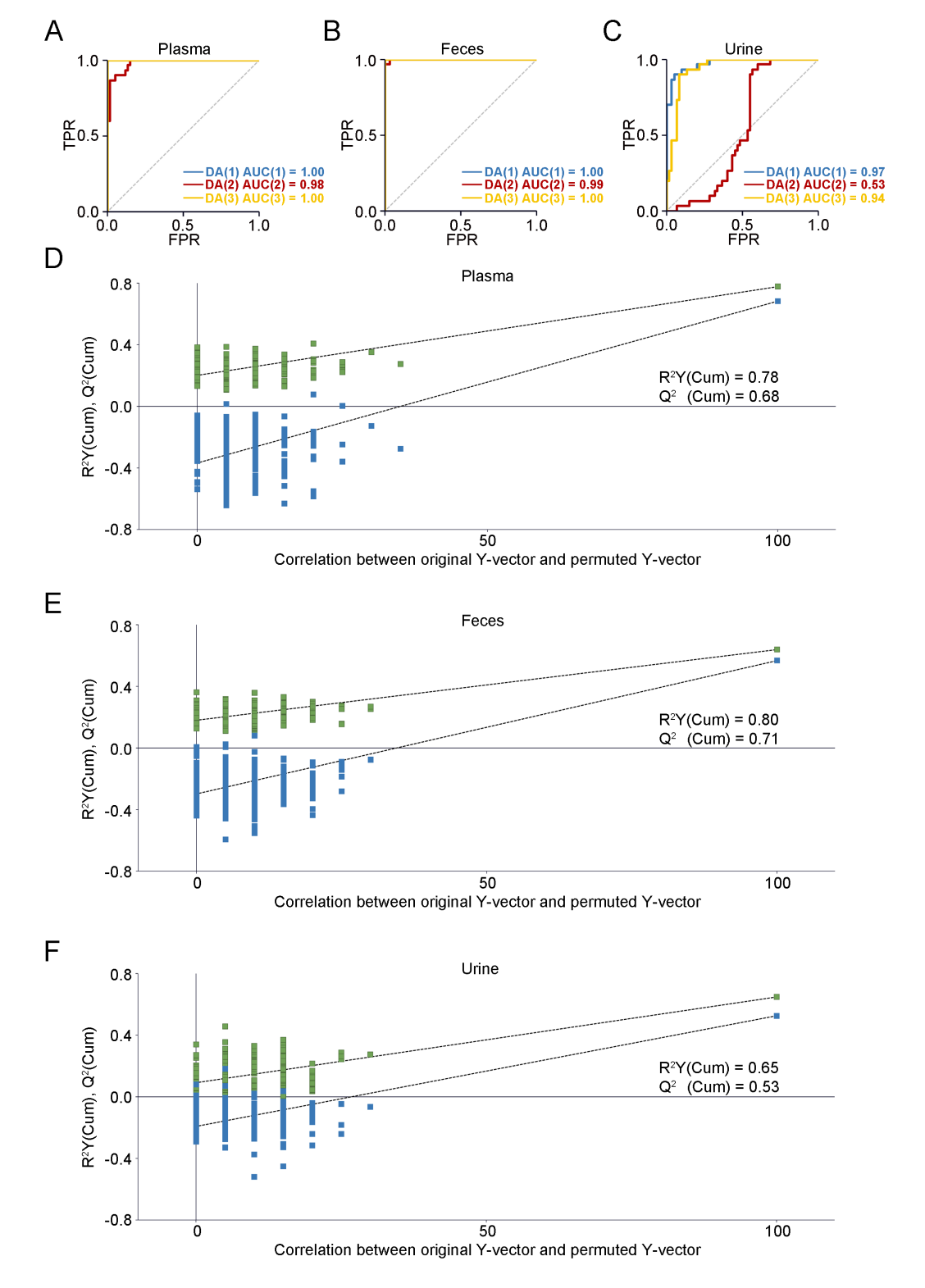


**Supplementary Figure 1. The validation of the OPLS-DA models.** The ROC analyses of the binary classifier of the OPLS-DA models in the **(A)** plasma, **(B)** feces and **(C)** urine. The permutation plots of the OPLS-DA models in the **(D)** plasma, **(E)** feces and **(F)** urine. The permutation indicates the correlation coefficient between the original R^2^(green dots), Q^2^ (blue dots), and cumulative R^2^, Y^2^; the dashed lines represent the corresponding regression lines. The number of random permutation tests for each plot was 200. OPLS-DA: Orthogonal partial-least-squares discriminant analysis; ROC: receiver operator characteristic; AUC: area under the ROC curve; TPR: ture positive rate; FPR: false positive rate.

**Supplementary Table 1.** The standard compounds of bile acids in our project.

| **MS No.*** | **BAs** | **Abbr.** | **HMDB ID** | **CAS No.** |
| --- | --- | --- | --- | --- |
| 1 | Cholic acid | CA | HMDB00619 | 81-25-4 |
| 2 | Chenodeoxycholic acid-3-β-D-glucuronide | CDCA-3Gln | HMDB02430 | 58814-71-4 |
| 3 | Glycochenodeoxycholic acid | GCDCA | HMDB00637 | 640-79-9 |
| 4 | Glycoursodeoxycholic acid | GUDCA | HMDB00708 | 64480-66-6 |
| 5 | Glycocholic acid | GCA | HMDB00138 | 475-31-0 |
| 6 | Glycolithocholic acid | GLCA | HMDB00698 | 474-74-8 |
| 7 | Taurochenodeoxycholic acid | TCDCA | HMDB00951 | 516-35-8 |
| 8 | Chenodeoxycholic acid | CDCA | HMDB00518 | 474-25-9 |
| 9 | Lithocholic acid | LCA | HMDB00761 | 434-13-9 |
| 10 | Taurocholic acid | TCA | HMDB00036 | 81-24-3 |
| 11 | 3β-Ursodeoxycholic Acid | 3β-UDCA | HMDB00686 | 78919-26-3 |
| 12 | 3β-deoxycholic acid | 3β-DCA | HMDB00438 | 570-63-8 |
| 13 | Glycohyocholic acid | GHCA | HMDB0240607 | 32747-08-3 |
| 14 | 3β-Cholic Acid | 3β-CA | HMDB00419 | 3338-16-7 |
| 15 | Glycolithocholic acid-3-sulfate | GLCA-3S | HMDB02639 | 15324-64-8 |
| 16 | Glycodeoxycholic acid | GDCA | HMDB00631 | 360-65-6 |
| 17 | Deoxycholic acid | DCA | HMDB00626 | 83-44-3 |
| 18 | Ursodeoxycholic acid | UDCA | HMDB00946 | 128-13-2 |
| 19 | Taurodeoxycholic acid | TDCA | HMDB00896 | 516-50-7 |
| 20 | Hyocholic acid | HCA | HMDB00760 | 547-75-1 |
| 21 | Norcholic acid | NCA | HMDB0255727 | 60696-62-0 |
| 22 | Taurolithocholic acid-3-sulfate | TLCA-3S | HMDB02580 | 15324-65-9 |
| 23 | Tauroursodeoxycholic acid | TUDCA | HMDB00874 | 14605-22-2 |
| 24 | Lithocholic acid-3-sulfate | LCA-3S | HMDB0000907 | 34669-57-3 |
| 25 | Isolithocholic acid | ILCA | HMDB00717 | 1534-35-6 |
| 26 | 3-oxodeoxycholic acid | 3-oxo-DCA | HMDB62742 | 4185-01-7 |
| 27 | Tauro-β-muricholic acid | Tβ-MCA | HMDB00932 | 25696-60-0 |
| 28 | ω-muricholic acid | ω-MCA | HMDB0000364 | 6830-03-1 |
| 29 | 7-ketolithocholic acid | 7-KLCA | HMDB00467 | 4651-67-6 |
| 30 | Taurolithocholic acid | TLCA | HMDB00722 | 516-90-5 |
| 31 | 7-Ketodeoxycholic acid | 7-KDCA | HMDB00391 | 911-40-0 |
| 32 | Ursocholic acid | UCA | HMDB00917 | 2955-27-3 |
| 33 | Dehydrocholic acid | DHCA | HMDB0304121 | 81-23-2 |
| 34 | Taurohyocholic acid | THCA | HMDB11637 | 117997-17-8 |
| 35 | 12-ketolithocholic acid | 12-KLCA | HMDB00328 | 5130-29-0 |
| 36 | 3-Oxocholic acid | 3-oxo-CA | HMDB00502 | 2304-89-4 |
| 37 | 12-Oxochenodeoxycholic acid | 12-oxo-CDCA | HMDB0000400 | 2458-08-4 |
| 38 | β-muricholic acid | β-MCA | HMDB00415 | 2393-59-1 |
| 39 | Nor-Deoxycholic Acid | 23-DCA | / | 53608-86-9 |
| 40 | β-Hyodeoxycholic Acid | 3β-HDCA | HMDB00664 | 570-84-3 |
| 41 | Dehydrolithocholic acid | DLCA | / | 1553-56-6 |
| 42 | Isoallolithocholic acid | IALCA | HMDB0000713 | 2276-93-9 |
| 43 | 6,7-diketolithocholic acid | 6,7-DKLCA | / | 1643669-23-1 |
| 44 | 7,12-diketolithocholic acid | 7,12-DKLCA | / | 517-33-9 |
| 45 | Glycodehydrocholic acid | GDHCA | / | 3415-45-0 |
| 46 | Hyodeoxycholic acid | HDCA | HMDB0000733 | 83-49-8 |
| 47 | Isodeoxycholic acid | IDCA | HMDB0002536 | 566-17-6 |
| 48 | Murideoxycholic acid | MDCA | HMDB0000811 | 668-49-5 |
| 49 | Taurodehydrocholic acid | TDHCA | HMDB0258743 | / |
| 50 | α-muricholic acid | α-MCA | HMDB0000506 | 2393-58-0 |

*MS No. corresponds to the chromatogram shown in Figure 1A. MS, mass spectrometry; BAs, bile acids; Abbr, abbreviation. HMDB, Human Metabolome Database.

**Supplementary Table 2.** Gradient program of the liquid chromatography mobile phase.

| **Total time (min)** | **Flow rate (μL/min)** | **A^a^ (v/v, %)** | **B^b^ (v/v, %)** |
| --- | --- | --- | --- |
| 0.0 | 350 | 95 | 5 |
| 0.5 | 350 | 60 | 40 |
| 4.5 | 350 | 50 | 50 |
| 7.5 | 350 | 25 | 75 |
| 10.0 | 350 | 5 | 95 |
| 12.0 | 350 | 5 | 95 |
| 14.0 | 350 | 95 | 5 |

^a^Solvent A, water containing 0.01% acetic acid (v/v) and 5 mmol/L ammonium acetate; ^b^Solvent B, acetonitrile with 0.01% acetic acid (v/v). Temperature, 40℃; injection volume, 3 μL.

**Supplementary Table 3.** The optimized mass spectrometry conditions for the detection of bile acids.

| Item | Parameter |
| --- | --- |
| Capillary voltage | 4500V in the positive ion mode |
| Source temperature | 550 °C |
| Curtain gas | 30 psi |
| Collision gas | 10 psi |
| Pressure for nebulization gas | 40 psi |
| Evaporization gas | 40 psi |
| Entrance potential | 50 V |
| Collision cell exit | 10 V |

**Supplementary Table 4.** The mass spectrometry parameters of the bile acids.

| **MS No.*** | **BAs** | **MRM transition** | **DP**  **(V)** | **CE**  **(V)** | **Range**  **(mg/L)** |  | **Plasma** | |  | **Urine** | |  | **Feces** | |
| --- | --- | --- | --- | --- | --- | --- | --- | --- | --- | --- | --- | --- | --- | --- |
|  |  |  |  |  |  |  | **RT**  **(min)** | **Linearity (R^2^)** |  | **RT**  **(min)** | **Linearity (R^2^)** |  | **RT**  **(min)** | **Linearity (R^2^)** |
| 1 | CA | 407.4→407.4 | 6 | 12 | 4-2000 |  | 8.59 | 0.99919 |  | 8.62 | 0.99299 |  | 8.62 | 0.99960 |
| 2 | CDCA-3Gln | 567.3→567.3 | 30 | 36 | 20-2000 |  | 4.95 | 0.99845 |  | 5.13 | 0.99408 |  | 5.19 | 0.99848 |
| 3 | GCDCA | 448.3→74 | 30 | 84 | 8-2000 |  | 6.33 | 0.99983 |  | 6.45 | 0.99601 |  | 6.64 | 0.99929 |
| 4 | GUDCA | 448.3→73.8 | 20 | 87 | 8-2000 |  | 3.38 | 0.99951 |  | 3.47 | 0.99744 |  | 3.49 | 0.99973 |
| 5 | GCA | 464.3→464.3 | 44 | 12 | 8-2000 |  | 3.45 | 0.99897 |  | 3.57 | 0.99798 |  | 3.58 | 0.99978 |
| 6 | GLCA | 432.3→74 | 22 | 79 | 4-2000 |  | 9.56 | 0.99350 |  | 9.56 | 0.99882 |  | 9.66 | 0.99586 |
| 7 | TCDCA | 498.3→79.8 | 45 | 147 | 8-2000 |  | 5.25 | 0.99971 |  | 5.44 | 0.99302 |  | 5.33 | 0.99725 |
| 8 | CDCA | 391.3→391.3 | 43 | 15 | 8-2000 |  | 10.13 | 0.99800 |  | 10.14 | 0.99359 |  | 10.14 | 0.99919 |
| 9 | LCA | 375.3→375.3 | 96 | 11 | 8-2000 |  | 11.13 | 0.99201 |  | 11.14 | 0.99879 |  | 11.12 | 0.99650 |
| 10 | TCA | 514.3→79.8 | 67 | 139 | 8-2000 |  | 3.00 | 0.99954 |  | 3.16 | 0.99952 |  | 3.04 | 0.99990 |
| 11 | 3β-UDCA | 391.3→391.3 | 43 | 15 | 4-2000 |  | 8.53 | 0.99910 |  | 8.49 | 0.99637 |  | 8.48 | 0.99378 |
| 12 | 3β-DCA | 391.3→391.3 | 43 | 15 | 2-2000 |  | 9.57 | 0.99982 |  | 9.55 | 0.99902 |  | 9.55 | 0.99696 |
| 13 | GHCA | 464.3→464.3 | 61 | 10 | 8-2000 |  | 2.64 | 0.99734 |  | 2.73 | 0.99512 |  | 2.72 | 0.99886 |
| 14 | 3β-CA | 407.4→407.4 | 40 | 20 | 4-2000 |  | 5.73 | 0.99969 |  | 5.76 | 0.99434 |  | 5.74 | 0.99866 |
| 15 | GLCA-3S | 512.3→512.3 | 45 | 11 | 8-2000 |  | 4.41 | 0.99913 |  | 4.57 | 0.99948 |  | 4.71 | 0.99959 |
| 16 | GDCA | 448.3→73.8 | 54 | 77 | 8-2000 |  | 7.08 | 0.99978 |  | 7.22 | 0.99957 |  | 7.42 | 0.99999 |
| 17 | DCA | 391.3→391.3 | 20 | 11 | 4-2000 |  | 10.26 | 0.99993 |  | 10.27 | 0.99877 |  | 10.26 | 0.99919 |
| 18 | UDCA | 391.3→391.3 | 11 | 16 | 8-2000 |  | 8.90 | 0.99782 |  | 8.88 | 0.99943 |  | 8.88 | 0.99123 |
| 19 | TDCA | 498.3→79.8 | 45 | 144 | 4-2000 |  | 5.96 | 0.99985 |  | 6.15 | 0.99166 |  | 6.07 | 0.99723 |
| 20 | HCA | 407.4→407.4 | 45 | 12 | 8-2000 |  | 7.90 | 0.99814 |  | 7.93 | 0.99113 |  | 7.91 | 0.99721 |
| 21 | NCA | 393.3→393.3 | 20 | 7 | 8-2000 |  | 5.99 | 0.99912 |  | 6.03 | 0.99270 |  | 6.02 | 0.99679 |
| 22 | TLCA-3S | 281.1→96.9 | 55 | 34 | 8-2000 |  | 2.99 | 0.99779 |  | 3.11 | 0.99375 |  | 2.97 | 0.99683 |
| 23 | TUDCA | 498.3→79.8 | 45 | 143 | 4-2000 |  | 2.82 | 0.99961 |  | 2.97 | 0.99894 |  | 2.86 | 0.99985 |
| 24 | LCA-3S | 455.3→97 | 52 | 105 | 8-2000 |  | 2.99 | 0.99779 |  | 9.17 | 0.99367 |  | 9.16 | 0.99880 |
| 25 | ILCA | 375.3→375.3 | 54 | 19 | 2-2000 |  | 10.87 | 0.99681 |  | 10.84 | 0.99894 |  | 10.83 | 0.99923 |
| 26 | 3-oxo-DCA | 389.3→345.3 | 40 | 37 | 8-2000 |  | 10.27 | 0.99942 |  | 10.28 | 0.99710 |  | 10.28 | 0.99973 |
| 27 | Tβ-MCA | 514.3→79.8 | 67 | 139 | 8-2000 |  | 1.90 | 0.99900 |  | 2.01 | 0.99724 |  | 1.90 | 0.99967 |
| 28 | ω-MCA | 407.4→407.4 | 58 | 10 | 8-2000 |  | 5.52 | 0.99958 |  | 5.53 | 0.99248 |  | 5.49 | 0.99947 |
| 29 | 7-KLCA | 389.3→389.3 | 22 | 18 | 8-2000 |  | 9.56 | 0.99834 |  | 9.56 | 0.99587 |  | 9.55 | 0.99750 |
| 30 | TLCA | 482.3→482.3 | 36 | 10 | 4-2000 |  | 8.73 | 0.99927 |  | 8.80 | 0.99556 |  | 8.78 | 0.99896 |
| 31 | 7-KDCA | 405.3→405.3 | 40 | 10 | 8-2000 |  | 6.33 | 0.99942 |  | 6.39 | 0.99557 |  | 6.39 | 0.99813 |
| 32 | UCA | 407.4→407.4 | 49 | 18 | 4-2000 |  | 4.23 | 0.99958 |  | 4.27 | 0.99815 |  | 4.22 | 0.99916 |
| 33 | DHCA | 401.3→401.3 | 61 | 11 | 8-2000 |  | 5.39 | 0.99942 |  | 5.41 | 0.99053 |  | 5.39 | 0.99960 |
| 34 | THCA | 514.3→79.8 | 67 | 139 | 8-2000 |  | 2.29 | 0.99981 |  | 2.41 | 0.99389 |  | 2.30 | 0.99975 |
| 35 | 12-KLCA | 389.3→389.3 | 22 | 16 | 8-2000 |  | 9.73 | 0.99902 |  | 9.73 | 0.99279 |  | 9.73 | 0.99853 |
| 36 | 3-oxo-CA | 405.3→405.3 | 16 | 9 | 8-2000 |  | 8.41 | 0.99933 |  | 8.45 | 0.99770 |  | 8.45 | 0.99816 |
| 37 | 12-oxo-CDCA | 405.3→405.3 | 60 | 11 | 8-2000 |  | 6.49 | 0.99918 |  | 6.55 | 0.99634 |  | 6.53 | 0.99770 |
| 38 | β-MCA | 407.4→407.4 | 40 | 20 | 4-2000 |  | 6.66 | 0.99964 |  | 6.64 | 0.99316 |  | 6.63 | 0.99773 |
| 39 | 23-DCA | 377.3→377.3 | 86 | 13 | 8-2000 |  | 9.65 | 0.99877 |  | 9.66 | 0.99684 |  | 9.65 | 0.99627 |
| 40 | 3β-HDCA | 391.3→391.3 | 25 | 11 | 8-2000 |  | 8.73 | 0.99947 |  | 8.69 | 0.99868 |  | 8.69 | 0.99995 |
| 41 | DLCA | 373.3→373.3 | 70 | 11 | 2-2000 |  | 11.21 | 0.99422 |  | 11.21 | 0.99089 |  | 11.20 | 0.99531 |
| 42 | IALCA | 376.3→59.0 | -96 | -11 | 8-2000 |  | 10.79 | 0.99642 |  | 10.75 | 0.99986 |  | 10.74 | 0.99796 |
| 43 | 6,7-DKLCA | 403.3→347.2 | -46 | -9 | 8-2000 |  | 9.67 | 0.99930 |  | 9.66 | 0.99258 |  | 9.66 | 0.99897 |
| 44 | 7,12-DKLCA | 403.3→385.2 | -46 | -9 | 8-2000 |  | 4.70 | 0.99834 |  | 4.73 | 0.99384 |  | 4.70 | 0.99927 |
| 45 | GDHCA | 458.3→74.0 | -18 | -83 | 8-2000 |  | 2.02 | 0.99614 |  | 2.11 | 0.99869 |  | 2.06 | 0.99911 |
| 46 | HDCA | 391.3→391.3 | -45 | -15 | 4-2000 |  | 8.98 | 0.99312 |  | 10.86 | 0.99822 |  | 8.98 | 0.99551 |
| 47 | IDCA | 391.3→391.3 | -20 | -11 | 4-2000 |  | 10.87 | 0.99624 |  | 10.86 | 0.99902 |  | 10.85 | 0.99939 |
| 48 | MDCA | 391.3→391.3 | -60 | -13 | 8-2000 |  | 8.36 | 0.99668 |  | 8.30 | 0.99654 |  | 8.30 | 0.99445 |
| 49 | TDHCA | 508.3→508.3 | -40 | -13 | 8-2000 |  | 1.84 | 0.99943 |  | 1.95 | 0.99622 |  | 1.85 | 0.99963 |
| 50 | α-MCA | 407.4→405.3 | -45 | -12 | 8-2000 |  | 6.06 | 0.99883 |  | 6.02 | 0.99660 |  | 6.01 | 0.99967 |

*MS No. corresponds to the chromatogram shown in Figure 1A. MS, mass spectrometry; BAs, bile acids; MRM, multiple reaction monitoring; DP, declustering potential; CE, collision energy; RT, retention time.

**Supplementary Table 5.** The mass spectrometry parameters of the isotoped-labelled bile acids.

| Compound | Abbreviation | DP (V) | CE (V) |
| --- | --- | --- | --- |
| Lithocholic acid-d4 | LCA-d4 | -60 | -12 |
| Chenodeoxycholic acid-d4 | CDCA-d4 | -50 | -12 |
| Deoxycholic acid-d4 | DCA-d4 | -67 | -9 |
| Cholic acid-d4 | CA-d4 | -19 | -10 |
| Glycolithocholic acid-d4 | GLCA-d4 | -25 | -85 |
| Glycochenodeoxycholic acid-d4 | GCDCA-d4 | -70 | -90 |
| Glycodeoxycholic acid-d4 | GDCA-d4 | -70 | -87 |
| Tauroursodeoxycholic-2,2,3,4,4-d5 Acid | TUDCA-d5 | -66 | -162 |
| Taurocholic acid-d4 | TCA-d4 | -29 | -124 |

DP, declustering potential; CE, collision energy

**Supplementary Table 6.** General characteristics of the study population

|  | **CON (n=30)** | **T2DM (n=30)** | **DKD (n=30)** | ***P*** |
| --- | --- | --- | --- | --- |
| Age, years | 58.50 ± 4.32 | 60.30 ± 7.04 | 60.37 ± 6.27 | 0.40 |
| Male/Female, n | 18/12 | 18/12 | 21/9 | 0.65 |
| Hb, g/L | 144.5 ± 8.6 | 130.6 ± 15.0 | 99.3 ± 21.7 | <0.01 |
| Glu, mM | 4.70 ± 0.50 | 9.08 ± 2.59 | 6.29 ± 2.83 | <0.01 |
| Alb, g/L | 47.96 ± 3.31 | 40.41 ± 4.51 | 35.28 ± 7.69 | <0.01 |
| Scr,mg/dL | 0.74 ± 0.11 | 0.74 ± 0.18 | 4.67 ± 3.14 | <0.01 |
| TC, mM | 4.40 ± 0.53 | 4.20 ± 1.13 | 5.06 ± 1.46 | <0.01 |
| TG, mM | 1.01 ± 0.36 | 1.82 ± 1.06 | 1.55 ± 0.85 | <0.01 |
| LDL-C, mM | 2.07 ± 0.68 | 2.60 ± 0.78 | 3.13 ± 1.49 | <0.01 |
| HDL-C, mM | 1.36 ± 0.42 | 1.84 ± 1.44 | 1.17 ± 0.37 | 0.59 |
| PTH, pg/mL | / | 31.07 ± 15.13 | 116.3 ± 85.5 | <0.01 |
| HbAlc, % | / | 6.72 ± 0.03 | 9.62 ± 2.02 | <0.01 |
| Urinary protein, g/24h | / | 0.11 ± 0.03 | 4.66 ± 3.10 | <0.01 |
| Urinary albumin,  Mg/24h | / | 16.28 ± 11.50 | 2365.97 ± 1847.57 | <0.01 |
| eGFR, mL/min/1.73m^2^ | 105.23 ± 15.19 | 96.27 ± 12.52 | 31.08 ± 30.20 | <0.01 |
| Comorbidity |  |  |  |  |
| CAD, n | / | 8 | 14 | 0.18 |
| HTN, n | / | 16 | 18 | 0.80 |
| DR, n | / | 11 | 11 | 1.00 |
| DPN, n | / | 3 | 14 | <0.01 |

PTH, HbAlc,24-hours urinary protein and 24-hours urinary albumin were not tested for healthy subjects in regular health examination. Continuous variables are presented as mean ± standard deviation and were compared with the Student’s t test. Categorical variables were compared by Fisher’s exact test and presented as counts. Hb, hemoglobin; Glu, glucose; Alb, albumin; TC, total cholesterol; TG, triglyceride; LDL-C, low density lipoprotein cholesterol; HDL-C, high density lipoprotein cholesterol; PTH, parathyroid hormone; HbAlc, glycated hemoglobin; UAER, urinary albumin excretion rate; eGFR, estimated glomerular filtration rate; CAD, coronary artery disease; HTN, hypertension; DR, diabetic retinopathy; DPN, diabetic peripheral neuropathy; CON, healthy subjects; T2DM, type 2 diabetes mellitus; DKD, diabetic kidney disease.

**Supplementary Table 7.** Plasma levels of the 27 bile acids in study participants.

| **BA**  **(mg/L)** | **CON**  **(n=30)** | **T2DM**  **(n=30)** | **DKD**  **(n=30)** | **F*** | ***P*^#^** | **DKD vs CON** | |  | **DKD vs T2DM** | |  | **DKD vs**  **T2DM + CON** | |  | **DKD + T2DM**  **vs CON** | |
| --- | --- | --- | --- | --- | --- | --- | --- | --- | --- | --- | --- | --- | --- | --- | --- | --- |
|  |  |  |  |  |  | **VIP** | ***P*^&^** |  | **VIP** | ***P*^&^** |  | **VIP** | ***P*^&^** |  | **VIP** | ***P*^&^** |
| CA | 43.65 ± 31.27 | 41.01 ± 25.82 | 45.92 ± 17.09 | 0.28 | 0.75 | 0.47 | 0.73 |  | 0.54 | 0.39 |  | 0.62 | 0.53 |  | 0.30 | 0.97 |
| CDCA-3Gln | 13.75 ± 11.77 | 29.77 ± 27.87 | 34.57 ± 17.77 | 8.69 | <0.01 | 1.40 | <0.01 |  | 0.91 | 0.43 |  | 0.99 | <0.01 |  | 1.12 | <0.01 |
| GCDCA | 263.98 ± 138.47 | 405.60 ± 193.47 | 1085.73 ± 531.47 | 51.23 | <0.01 | 1.77 | <0.01 |  | 1.89 | <0.01 |  | 2.04 | <0.01 |  | 1.69 | <0.01 |
| GUDCA | 24.49 ± 13.22 | 23.22 ± 18.14 | 32.09 ± 18.54 | 2.44 | 0.09 | 0.89 | 0.07 |  | 0.89 | 0.07 |  | 0.86 | 0.03 |  | 0.88 | 0.41 |
| GCA | 53.56 ± 29.50 | 73.06 ± 32.82 | 155.71 ± 85.80 | 28.43 | <0.01 | 1.68 | <0.01 |  | 1.70 | <0.01 |  | 1.88 | <0.01 |  | 1.70 | <0.01 |
| GLCA | 7.15 ± 5.24 | 7.54 ± 4.78 | 6.98 ± 3.76 | 0.12 | 0.89 | 0.55 | 0.88 |  | 0.65 | 0.62 |  | 0.65 | 0.72 |  | 0.44 | 0.92 |
| TCDCA | 40.47 ± 23.04 | 83.31 ± 44.61 | 72.05 ± 41.13 | 10.54 | <0.01 | 1.31 | <0.01 |  | 0.92 | 0.31 |  | 0.96 | 0.27 |  | 1.38 | <0.01 |
| CDCA | 673.56 ± 263.11 | 635.52 ± 263.38 | 609.90 ± 259.10 | 0.45 | 0.64 | 0.51 | 0.35 |  | 0.55 | 0.71 |  | 0.44 | 0.45 |  | 0.39 | 0.39 |
| LCA | 6.43 ± 3.45 | 7.56 ± 3.51 | 7.43 ± 3.68 | 0.90 | 0.41 | 0.62 | 0.29 |  | 0.74 | 0.89 |  | 0.59 | 0.59 |  | 0.37 | 0.18 |
| TCA | 7.08 ± 5.87 | 10.52 ± 6.01 | 21.40 ± 11.97 | 23.51 | <0.01 | 1.50 | <0.01 |  | 1.60 | <0.01 |  | 1.71 | <0.01 |  | 1.42 | <0.01 |
| 3β-UDCA | 83.69 ± 40.16 | 91.89 ± 36.69 | 79.75 ± 46.22 | 0.68 | 0.51 | 0.28 | 0.73 |  | 0.51 | 0.26 |  | 0.42 | 0.38 |  | 0.06 | 0.82 |
| 3β-DCA | 39.29 ± 24.37 | 61.48 ± 23.37 | 57.71 ± 28.11 | 6.57 | <0.01 | 0.89 | <0.01 |  | 0.19 | 0.57 |  | 0.51 | 0.23 |  | 1.08 | <0.01 |
| GHCA | 3.16 ± 2.32 | 8.90 ± 3.61 | 15.17 ± 7.24 | 45.94 | <0.01 | 1.77 | <0.01 |  | 1.52 | <0.01 |  | 1.84 | <0.01 |  | 1.92 | <0.01 |
| 3β-CA | 3.81 ± 2.64 | 3.36 ± 3.15 | 3.83 ± 2.38 | 0.28 | 0.76 | 0.42 | 0.97 |  | 0.59 | 0.52 |  | 0.58 | 0.69 |  | 0.60 | 0.73 |
| GLCA-3S | 168.36 ± 93.76 | 192.86 ± 84.46 | 159.75 ± 79.47 | 1.19 | 0.31 | 0.84 | 0.70 |  | 0.90 | 0.12 |  | 0.86 | 0.28 |  | 0.33 | 0.68 |
| GDCA | 227.24 ± 95.40 | 153.25 ± 62.72 | 200.66 ± 114.12 | 4.85 | 0.01 | 0.89 | 0.33 |  | 1.08 | 0.05 |  | 0.68 | 0.63 |  | 1.25 | 0.02 |
| DCA | 161.30 ± 89.50 | 208.34 ± 76.27 | 153.81 ± 103.54 | 3.20 | 0.05 | 0.39 | 0.77 |  | 1.03 | 0.02 |  | 0.59 | 0.14 |  | 0.27 | 0.34 |
| UDCA | 68.42 ± 47.46 | 91.17 ± 35.94 | 58.87 ± 45.22 | 4.44 | 0.02 | 0.38 | 0.43 |  | 1.39 | 0.01 |  | 0.91 | 0.04 |  | 0.24 | 0.51 |
| TDCA | 27.33 ± 14.22 | 15.07 ± 9.22 | 19.07 ± 14.48 | 7.08 | <0.01 | 0.86 | 0.03 |  | 0.79 | 0.21 |  | 0.28 | 0.49 |  | 1.30 | <0.01 |
| HCA | 9.51 ± 5.29 | 10.87 ± 4.94 | 10.85 ± 7.73 | 0.49 | 0.62 | 0.87 | 0.44 |  | 0.80 | 0.99 |  | 0.86 | 0.63 |  | 0.78 | 0.32 |
| NCA | 5.23 ± 2.47 | 3.10 ± 1.75 | 2.75 ± 1.75 | 13.33 | <0.01 | 1.30 | <0.01 |  | 0.58 | 0.45 |  | 0.98 | <0.01 |  | 1.59 | <0.01 |
| TLCA-3S | 31.52 ± 25.33 | 26.14 ± 15.20 | 35.73 ± 20.87 | 1.59 | 0.21 | 0.59 | 0.49 |  | 1.01 | 0.05 |  | 0.68 | 0.14 |  | 0.67 | 0.90 |
| TUDCA | 0.9 ± 0.88 | 1.07 ± 0.77 | 1.49 ± 1.24 | 2.84 | 0.06 | 0.91 | 0.04 |  | 1.01 | 0.12 |  | 0.98 | 0.02 |  | 0.98 | 0.09 |
| LCA-3S | 5.63 ± 4.62 | 6.89 ± 3.75 | 5.58 ± 3.39 | 1.05 | 0.35 | 0.50 | 0.97 |  | 0.91 | 0.16 |  | 0.76 | 0.45 |  | 0.36 | 0.50 |
| ILCA | 20.62 ± 11.84 | 21.46 ± 11.54 | 23.30 ± 8.24 | 0.50 | 0.61 | 0.69 | 0.31 |  | 0.51 | 0.48 |  | 0.59 | 0.34 |  | 0.34 | 0.46 |
| 3-oxo-DCA | 4.54 ± 2.92 | 5.79 ± 2.49 | 5.46 ± 3.81 | 1.30 | 0.28 | 1.98 | 0.30 |  | 0.64 | 0.69 |  | 0.73 | 0.68 |  | 0.55 | 0.12 |
| Tβ-MCA | 1.34 ± 0.99 | 1.87 ± 1.49 | 2.76 ± 2.33 | 5.43 | <0.01 | 1.07 | <0.01 |  | 0.94 | 0.08 |  | 1.08 | <0.01 |  | 0.97 | 0.01 |

Data represent mean ± standard deviation. F* values and *P*^#^ values were determined by one-way analysis of variance. VIP values were determined based on orthogonal partical least squares discriminant analysis. *P*^&^ values were determined by Student’s t-test. CON, healthy controls; T2DM, type 2 diabetes mellitus; DKD, diabetic kidney disease; VIP, variable importance in projection.

**Supplementary Table 8.** Fecal levels of the 30 bile acids in study participants.

| **BA**  **(mg/g)** | **CON**  **(n=30)** | **T2DM**  **(n=30)** | **DKD**  **(n=30)** | **F*** | ***P*^#^** | **DKD vs CON** | |  | **DKD vs T2DM** | |  | **DKD vs**  **T2DM + CON** | |  | **DKD + T2DM**  **vs CON** | |
| --- | --- | --- | --- | --- | --- | --- | --- | --- | --- | --- | --- | --- | --- | --- | --- | --- |
|  |  |  |  |  |  | **VIP** | ***P*^&^** |  | **VIP** | ***P*^&^** |  | **VIP** | ***P*^&^** |  | **VIP** | ***P*^&^** |
| CA | 23.06 ± 13.61 | 75.91 ± 58.53 | 36.16 ± 33.66 | 14.38 | <0.01 | 0.88 | 0.05 |  | 1.20 | <0.01 |  | 0.74 | 0.19 |  | 1.44 | <0.01 |
| CDCA-3Gln | 0.36 ± 0.23 | 0.26 ± 0.11 | 0.76 ± 0.44 | 25.01 | <0.01 | 1.26 | <0.01 |  | 1.80 | <0.01 |  | 1.71 | <0.01 |  | 0.81 | 0.07 |
| DCA | 263.32 ± 76.21 | 243.02 ± 95.50 | 276.24 ± 81.54 | 1.17 | 0.32 | 0.39 | 0.53 |  | 0.83 | 0.15 |  | 0.65 | 0.23 |  | 0.53 | 0.85 |
| GCDCA | 5.39 ± 3.43 | 4.74 ± 3.20 | 4.52 ± 1.53 | 0.75 | 0.47 | 0.34 | 0.21 |  | 0.12 | 0.73 |  | 0.23 | 0.39 |  | 0.57 | 0.23 |
| GLCA | 0.07 ± 0.04 | 0.05 ± 0.03 | 0.20 ± 0.10 | 54.15 | <0.01 | 1.64 | <0.01 |  | 2.02 | <0.01 |  | 2.05 | <0.01 |  | 1.20 | <0.01 |
| GUDCA | 0.63 ± 0.39 | 0.50 ± 0.43 | 0.38 ± 0.22 | 3.72 | 0.023 | 0.97 | <0.01 |  | 0.42 | 0.19 |  | 0.66 | 0.02 |  | 1.08 | 0.02 |
| LCA | 476.55 ± 187.58 | 493.65 ± 285.73 | 540.76 ± 238.45 | 0.57 | 0.57 | 0.84 | 0.25 |  | 0.82 | 0.49 |  | 0.89 | 0.30 |  | 0.74 | 0.45 |
| NCA | 0.97 ± 0.52 | 0.98 ± 0.63 | 1.18 ± 0.57 | 1.27 | 0.29 | 0.38 | 0.14 |  | 0.71 | 0.21 |  | 0.59 | 0.11 |  | 0.47 | 0.39 |
| GCA | 6.48 ± 4.03 | 1.68 ± 1.14 | 5.11 ± 3.28 | 19.50 | <0.01 | 0.39 | 0.15 |  | 1.69 | <0.01 |  | 0.43 | 0.21 |  | 1.27 | <0.01 |
| 7-KDCA | 192.64 ± 154.35 | 203.69 ± 145.68 | 516.54 ± 277.49 | 24.94 | <0.01 | 1.44 | <0.01 |  | 1.46 | <0.01 |  | 1.70 | <0.01 |  | 1.23 | <0.01 |
| TCA | 5.81 ± 3.26 | 4.38 ± 2.91 | 3.51 ± 3.06 | 4.27 | 0.02 | 0.87 | <0.01 |  | 0.60 | 0.27 |  | 0.77 | 0.03 |  | 1.04 | <0.01 |
| CDCA | 215.28 ± 93.85 | 318.13 ± 280.11 | 66.23 ± 55.51 | 15.98 | <0.01 | 1.70 | <0.01 |  | 1.38 | <0.01 |  | 1.25 | <0.01 |  | 0.76 | 0.61 |
| GDCA | 3.08 ± 2.32 | 2.87 ± 2.16 | 3.69 ± 2.54 | 0.98 | 0.38 | 0.46 | 0.34 |  | 0.79 | 0.19 |  | 0.68 | 0.18 |  | 0.47 | 0.71 |
| LCA-3S | 1.90 ± 0.86 | 4.09 ± 4.01 | 6.28 ± 5.25 | 9.77 | <0.01 | 1.22 | <0.01 |  | 0.89 | 0.07 |  | 1.11 | <0.01 |  | 1.13 | <0.01 |
| ω-MCA | 0.82 ± 0.39 | 0.89 ± 0.59 | 1.60 ± 0.82 | 14.11 | <0.01 | 1.29 | <0.01 |  | 1.21 | <0.01 |  | 1.39 | <0.01 |  | 1.00 | <0.01 |
| 3-oxo-DCA | 80.70 ± 37.11 | 98.90 ± 58.14 | 103.74 ± 51.02 | 1.81 | 0.17 | 0.59 | 0.05 |  | 0.65 | 0.73 |  | 0.55 | 0.21 |  | 0.42 | 0.06 |
| TCDCA | 0.86 ± 0.53 | 0.55 ± 0.42 | 0.57 ± 0.39 | 4.31 | 0.02 | 1.08 | 0.02 |  | 0.71 | 0.82 |  | 0.87 | 0.22 |  | 1.26 | <0.01 |
| 3β-HDCA | 0.96 ± 0.72 | 0.92 ± 0.68 | 1.74 ± 1.35 | 6.89 | <0.01 | 0.94 | <0.01 |  | 0.98 | <0.01 |  | 1.12 | <0.01 |  | 0.75 | 0.11 |
| 7-KLCA | 23.06 ± 20.22 | 42.42 ± 35.63 | 37.54 ± 32.09 | 3.37 | 0.04 | 1.06 | 0.04 |  | 0.91 | 0.58 |  | 0.95 | 0.49 |  | 1.25 | 0.01 |
| TUDCA | 0.35 ± 0.26 | 0.22 ± 0.15 | 0.18 ± 0.13 | 6.87 | <0.01 | 1.12 | <0.01 |  | 0.94 | 0.27 |  | 1.01 | 0.02 |  | 1.32 | <0.01 |
| HCA | 5.78 ± 4.39 | 4.53 ± 3.66 | 4.44 ± 2.99 | 1.22 | 0.30 | 0.98 | 0.17 |  | 0.65 | 0.92 |  | 0.83 | 0.40 |  | 0.84 | 0.12 |
| TDCA | 0.67 ± 0.44 | 0.18 ± 0.12 | 0.24 ± 0.14 | 28.61 | <0.01 | 1.49 | <0.01 |  | 0.62 | 0.12 |  | 0.85 | 0.01 |  | 2.09 | <0.01 |
| 3β-UDCA | 8.88 ± 6.26 | 9.64 ± 6.26 | 6.65 ± 4.32 | 2.25 | 0.11 | 0.89 | 0.11 |  | 0.76 | 0.04 |  | 0.77 | 0.04 |  | 0.77 | 0.57 |
| HDCA | 27.47 ± 20.66 | 25.47 ± 17.13 | 25.11 ± 23.89 | 0.11 | 0.89 | 0.27 | 0.68 |  | 0.26 | 0.95 |  | 0.30 | 0.77 |  | 0.27 | 0.64 |
| 12-KLCA | 66.14 ± 27.64 | 77.01 ± 43.22 | 95.91 ± 39.98 | 4.83 | 0.01 | 0.99 | <0.01 |  | 0.73 | 0.08 |  | 0.90 | <0.01 |  | 0.82 | 0.02 |
| 3β-DCA | 126.92 ± 54.33 | 126.56 ± 69.44 | 150.09 ± 69.65 | 1.30 | 0.28 | 0.59 | 0.16 |  | 0.79 | 0.20 |  | 0.76 | 0.11 |  | 0.53 | 0.44 |
| DLCA | 86.98 ± 44.61 | 92.85 ± 54.00 | 107.69 ± 47.37 | 1.43 | 0.24 | 0.65 | 0.09 |  | 0.56 | 0.26 |  | 0.69 | 0.11 |  | 0.70 | 0.23 |
| ILCA | 99.60 ± 71.72 | 156.62 ± 117.16 | 277.47 ± 171.63 | 15.36 | <0.01 | 1.51 | <0.01 |  | 1.19 | <0.01 |  | 1.49 | <0.01 |  | 1.46 | <0.01 |
| IALCA | 11.17 ± 9.21 | 8.60 ± 6.17 | 11.74 ± 9.78 | 1.15 | 0.32 | 0.67 | 0.82 |  | 0.74 | 0.14 |  | 0.63 | 0.34 |  | 0.64 | 0.60 |
| MDCA | 0.55 ± 0.29 | 0.43 ± 0.28 | 0.53 ± 0.39 | 1.04 | 0.36 | 0.63 | 0.80 |  | 0.77 | 0.30 |  | 0.78 | 0.64 |  | 0.81 | 0.35 |

Data represent mean ± standard deviation. F* values and *P*^#^ values were determined by one-way analysis of variance. VIP values were determined based on orthogonal partical least squares discriminant analysis. *P*^&^ values were determined by Student’s t-test. CON, healthy controls; T2DM, type 2 diabetes mellitus; DKD, diabetic kidney disease; VIP, variable importance in projection.

| **BA**  **(mg/L)** | **CON**  **(n=30)** | **T2DM**  **(n=30)** | **DKD**  **(n=30)** | **F*** | ***P*^#^** | **DKD vs CON** | |  | **DKD vs T2DM** | |  | **DKD vs**  **T2DM + CON** | |  | **DKD + T2DM**  **vs CON** | |
| --- | --- | --- | --- | --- | --- | --- | --- | --- | --- | --- | --- | --- | --- | --- | --- | --- |
|  |  |  |  |  |  | **VIP** | ***P*^&^** |  | **VIP** | ***P*^&^** |  | **VIP** | ***P*^&^** |  | **VIP** | ***P*^&^** |
| CA | 22.65 ± 12.94 | 23.74 ± 15.14 | 25.75 ± 14.39 | 0.37 | 0.69 | 0.52 | 0.38 |  | 0.41 | 0.60 |  | 0.45 | 0.42 |  | 0.40 | 0.51 |
| CDCA-3Gln | 141.79 ± 80.86 | 108.73 ± 55.67 | 123.65 ± 66.32 | 1.79 | 0.17 | 0.91 | 0.35 |  | 1.02 | 0.34 |  | 0.92 | 0.93 |  | 0.89 | 0.10 |
| DCA | 5.05 ± 2.70 | 4.63 ± 2.83 | 4.30 ± 2.60 | 0.58 | 0.56 | 0.58 | 0.28 |  | 1.15 | 0.65 |  | 0.71 | 0.38 |  | 0.71 | 0.33 |
| GCDCA | 5.95 ± 3.26 | 5.17 ± 2.82 | 4.69 ± 3.02 | 1.31 | 0.28 | 0.49 | 0.13 |  | 0.43 | 0.52 |  | 0.44 | 0.20 |  | 0.49 | 0.14 |
| GLCA | 0.55 ± 0.30 | 0.70 ± 0.49 | 0.76 ± 0.37 | 2.20 | 0.12 | 0.91 | 0.02 |  | 0.81 | 0.60 |  | 0.78 | 0.14 |  | 0.75 | 0.05 |
| GUDCA | 5.17 ± 3.50 | 4.61 ± 2.96 | 5.22 ± 2.75 | 0.36 | 0.70 | 0.81 | 0.95 |  | 0.81 | 0.41 |  | 0.82 | 0.63 |  | 0.77 | 0.71 |
| LCA | 1.10 ± 0.50 | 1.17 ± 0.71 | 0.98 ± 0.62 | 0.73 | 0.49 | 0.52 | 0.39 |  | 0.99 | 0.28 |  | 0.49 | 0.25 |  | 0.33 | 0.81 |
| NCA | 50.53 ± 27.73 | 51.40 ± 28.97 | 45.88 ± 22.26 | 0.38 | 0.69 | 0.90 | 0.48 |  | 0.80 | 0.41 |  | 0.80 | 0.39 |  | 0.93 | 0.75 |
| UCA | 144.11 ± 77.38 | 141.91 ± 87.71 | 135.62 ± 75.74 | 0.09 | 0.91 | 0.27 | 0.67 |  | 0.52 | 0.77 |  | 0.39 | 0.68 |  | 0.15 | 0.77 |
| GCA | 24.37 ± 14.27 | 23.15 ± 14.36 | 20.11 ± 11.76 | 0.79 | 0.46 | 0.75 | 0.21 |  | 0.46 | 0.37 |  | 0.59 | 0.23 |  | 0.69 | 0.37 |
| GLCA-3S | 332.02 ± 176.89 | 186.51 ± 96.59 | 87.97 ± 41.30 | 32.05 | <0.01 | 2.06 | <0.01 |  | 2.36 | <0.01 |  | 2.14 | <0.01 |  | 2.15 | <0.01 |
| 3β-CA | 10.42 ± 6.86 | 11.21 ± 8.33 | 8.36 ± 4.96 | 1.38 | 0.26 | 0.72 | 0.19 |  | 1.11 | 0.11 |  | 0.68 | 0.11 |  | 0.66 | 0.68 |
| 7-KDCA | 73.83 ± 45.36 | 30.23 ± 21.12 | 22.62 ± 14.61 | 23.30 | <0.01 | 1.86 | <0.01 |  | 1.55 | <0.01 |  | 1.94 | <0.01 |  | 1.91 | <0.01 |
| TLCA-3S | 149.05 ± 85.53 | 56.50 ± 35.06 | 35.46 ± 13.99 | 37.60 | <0.01 | 2.06 | <0.01 |  | 1.44 | <0.01 |  | 1.91 | <0.01 |  | 2.12 | <0.01 |
| TCA | 3.12 ± 1.90 | 2.17 ± 1.34 | 2.43 ± 1.43 | 2.96 | 0.06 | 1.04 | 0.11 |  | 0.87 | 0.47 |  | 0.99 | 0.55 |  | 1.14 | 0.02 |
| Tβ-MCA | 2.36 ± 1.37 | 2.32 ± 1.52 | 2.48 ± 1.44 | 0.10 | 0.91 | 0.15 | 0.74 |  | 0.27 | 0.68 |  | 0.32 | 0.66 |  | 0.29 | 0.89 |
| CDCA | 5.45 ± 2.94 | 5.71 ± 3.29 | 4.51 ± 2.75 | 1.32 | 0.27 | 0.92 | 0.21 |  | 0.89 | 0.13 |  | 0.77 | 0.11 |  | 0.57 | 0.62 |
| 3-oxo-CA | 4.71 ± 2.71 | 4.73 ± 3.12 | 4.11 ± 2.58 | 0.46 | 0.63 | 0.51 | 0.39 |  | 0.57 | 0.41 |  | 0.52 | 0.34 |  | 0.21 | 0.65 |
| GDCA | 5.46 ± 2.81 | 4.90 ± 2.91 | 5.23 ± 2.34 | 0.32 | 0.72 | 0.61 | 0.73 |  | 0.59 | 0.63 |  | 0.83 | 0.93 |  | 0.77 | 0.52 |
| 12-oxo-CDCA | 46.73 ± 25.13 | 40.79 ± 26.18 | 46.85 ± 22.03 | 0.60 | 0.55 | 0.45 | 0.98 |  | 0.68 | 0.34 |  | 0.63 | 0.57 |  | 0.47 | 0.60 |
| LCA-3S | 5.46 ± 2.44 | 5.30 ± 2.72 | 5.96 ± 2.83 | 0.50 | 0.61 | 0.92 | 0.46 |  | 1.14 | 0.36 |  | 1.06 | 0.33 |  | 0.97 | 0.77 |

**Supplementary Table 9.** Urine levels of the 21 bile acids in study participants.

Data represent mean ± standard deviation. F* values and *P*^#^ values were determined by one-way analysis of variance. VIP values were determined based on orthogonal partial least squares discriminant analysis. *P*^&^ values were determined by Student’s t-test. CON, healthy controls; T2DM, type 2 diabetes mellitus; DKD, diabetic kidney disease; VIP, variable importance in projection.
